# Hybrid genome assemblies of four Shiga toxin-producing *Escherichia coli* strains containing a complete locus of enterocyte effacement and an O-Island 122

**DOI:** 10.1128/MRA.00046-23

**Published:** 2023-10-31

**Authors:** Siobhán C. McCarthy, Guerrino Macori, Catherine M. Burgess, Geraldine Duffy, Séamus Fanning

**Affiliations:** 1 UCD-Center for Food Safety, School of Health, Physiotherapy and Sports Science, University College Dublin, Belfield, Dublin, Ireland; 2 Food Safety Department, Teagasc Food Research Center, Ashtown, Dublin, Ireland; 3 School of Biology and Environmental Science, University College Dublin, Dublin, Ireland; University of Maryland School of Medicine, Baltimore, Maryland, USA

**Keywords:** shiga toxin-producing* Escherichia coli*, foodborne disease, pathogenicity islands, next generation sequencing

## Abstract

This study describes the hybrid genome assemblies of four Shiga toxin-producing *Escherichia coli* strains isolated from the recto-anal junction of slaughter-age Irish sheep. *In silico* serotyping and genome analysis determined that each of the strains harbored a Shiga-toxin subtype, a complete locus of enterocyte effacement, and a rare O-island 122.

## ANNOUNCEMENT

Shiga toxin-producing *Escherichia coli* (STEC) are significant foodborne zoonotic pathogens with primary virulence determinants being the bacteriophage encoded Shiga toxins (Stx) and the intimin adhesion protein (1, 2). Auxiliary virulence factors on genomic pathogenicity islands determine an isolate’s disease potential (3 – 6), notably the locus of enterocyte effacement (LEE) and O-island (OI)-122 (7 – 11). We previously described the isolation of 178 STEC strains from Irish slaughter-age sheep (12). Genomic characterization revealed that four of these strains harbored a LEE locus and components of OI-122, factors that are rarely observed in STEC isolated from sheep. To further define these pathogenicity islands and genomic regions, we used a hybrid assembly approach for each of the four genomes

Recto-anal swab samples were collected from slaughter-age sheep at two slaughtering facilities. Swabs were enriched in 30 mL of prewarmed modified tryptone-soy broth (mTSB) with 16 mg/L of novobiocin (Oxoid) and incubated at 41.5°C for 24 h. RT-PCR was performed on DNA extracted using InstaGene matrix (Bio-Rad) as described by Macori et al. (13) to confirm the presence of Stx genes using primers and amplification conditions from McCarthy et al. (12). RT-PCR positive samples were plated on MacConkey agar (Oxoid) and incubated overnight (18 h) at 37°C and colonies were confirmed via RT-PCR and sub-cultured on Tryptone Soya Agar (Oxoid). Single colonies were grown overnight (18 h) in Tryptone Soy Broth (Oxoid) at 37°C with shaking (180 rpm) for both Illumina and Oxford Nanopore Technologies (ONT) genomic DNA preparations. DNA for short-read sequencing (Illumina) was extracted using the DNeasy UltraClean microbial kit (Qiagen), sequencing libraries were prepared with NEBNext Ultra II library preparation kit (New England Biolabs), and paired-end sequencing was performed on the MiSeq platform (Illumina) with a v3 chemistry paired-end (2 × 300 cylces). Templated DNA for ONT sequencing was extracted from the same overnight culture using the Wizard gDNA Kit (Promega), and libraries were constructed using the SQK-RAD004 rapid sequencing kit (ONT). Sequencing was carried out on MinION platform using Flongle flow cells (R9.4.1), and reads were basecalled with Guppy (v5.0.11). ONT and Illumina raw reads were quality assessed using FastQC (v0.11.9; https://www.bioinformatics.babraham.ac.uk/projects/fastqc) and trimmed using Porechop (v0.2.4) for ONT or Fastp (v0.20.1) (14) for Illumina outputs. Genome *de novo* hybrid assemblies were constructed with Unicycler (v0.4.9) (15) and ranged from 6 to 27 contigs with mean G + C content of 50.62% (Table 1).

**TABLE 1:** Genome assembly details and statistics

Strain	Serotype^a^	Shiga-toxin subtype^a^	OI-122	No. of Illumina reads	No. of MinION reads	MinION read *N*_50_ (bp)	Genome size (bp)	Assembly *N*_50_ (bp)	No. of contigs	No. of CDS	NCBI accession number (Minion, Illumina)	GenBank accession number	SRA accession number (Minion, Illumina)
VTEC12I	O157:H7	*stx_2c_*	*espL2, nleB, nleE*	1,095,718	54,699	11,053	5,591,504	5,494,962	6	5,385	SAMN20192600	JAHVRY000000000	SRX13417612, SRX11424687
VTEC20F	O145:H28	*stx_2a_*	*espL2, nleB, nleE*	939,240	60,563	12,014	5,692,504	3,700,680	22	5,483	SAMN20192538	JAHVUH000000000	SRX13417609, SRX11424650
VTEC9E	O5:H9	*stx_1a_*	*espL2, nleB, nleE, efa1*	896,695	38,343	8,843	5,363,230	3,685,233	22	5,252	SAMN20192558	JAHVTN000000000	SRX13417611, SRX11424671
VTEC9G	O103:H2	*stx_2a_*	*espL2, nleB, nleE*	1,114,186	22,501	8,563	5,799,655	2,039,782	27	5,681	SAMN20192540	JAHVUF000000000	SRX13417610, SRX11424732

^a^ Genome annotation including serotyping and Shiga toxin subtyping was performed as described by McCarthy et al. (12).

Genome annotation was conducted using the ABRIcate tool (v1.0.1) (16), and virulence factors were identified by interrogating the ecoli_vf database (https://github.com/phac-nml/ecoli_vf) and a customized database containing *nle* gene sequences (https://github.com/macgenomegue/nle_database). Protein-coding sequences, tRNAs, and insertion sequence elements were predicted using Prokka (1.14.6) (17), tRNA-scan-SE (v2.0.9) (18), and ISEScan (v1.7.2.3) (19), respectively, while bacteriophages were classified using the PHASTER (20) webtool. Standard parameters were used for all software.

## Data Availability

The raw sequence files and hybrid genome assemblies have been deposited in both the Sequence Read Archive and GenBank under the BioProject accession number PRJNA744868. The accession numbers for both repositories are listed in Table 1. The genome assemblies described in this paper are the third version.
